# The PREVCOG study: longitudinal and multimodal biomarker analyses for secondary prevention of Alzheimer's disease and cognitive decline in French memory clinics

**DOI:** 10.1002/alz70856_104308

**Published:** 2025-12-24

**Authors:** Germain Busto, Cédric Turpinat, Sophie Navucet, Emmanuelle Le Bars, Yves Dauvilliers, Julien Delrieu, Florence Perrein, Snejana Jurici, Sylvie Claeysen, Audrey Gabelle, Sylvain Lehmann, Karim Bennys

**Affiliations:** ^1^ CHU Montpellier CMRR, Montpellier, France; ^2^ Université de Montpellier, Memory Research and Resources center, department of Neurology, Inserm INM, F‐34000 Montpellier, France., Montpellier, France; ^3^ Memory Resources and Research Center, CHRU Gui de Chauliac, Montpellier, France; ^4^ Institut d'Imagerie Fonctionnelle Humaine, I2FH, CHRU Gui de Chauliac, Montpellier, France; ^5^ National Reference Centre for Orphan Diseases, Narcolepsy‐ Rare hypersomnias, CHU Montpellier, Montpellier, France; ^6^ Maintain Aging Research team, CERPOP, Inserm, Université Paul Sabatier, Toulouse, France; ^7^ CHU Nimes, Nimes, France; ^8^ CH Perpignan, Gériatrie, Perpignan, France; ^9^ Institut de Génomique Fonctionnelle (IGF), Montpellier, France; ^10^ Memory Resource and Research Center of Montpellier, CHU de Montpellier, Hôpital Gui de Chauliac, Montpellier, France; ^11^ LBPC‐PPC, Université de Montpellier, IRMB CHU de Montpellier, INM INSERM, Montpellier, Occitanie, France

## Abstract

**Background:**

While research on Alzheimer's disease has recently made significant progress with the approval of two disease‐modifying treatments and the development of blood‐based biomarkers predicted to revolutionize AD diagnosis, questions remain—particularly regarding the value of amyloidopathy in predicting cognitive decline on its own. The medical and scientific community continues to debate the relevance of amyloidopathy in prescribing anti‐amyloid treatments, especially considering the side effects and the fact that not all amyloid‐positive individuals will experience cognitive decline. Clearly, more data is needed to accurately predict cognitive decline and support patient‐centered clinical decisions.

**Method:**

Our objective is to longitudinally follow a cohort of cognitively unimpaired, amyloid‐positive individuals who have been screened using the most up‐to‐date blood biomarker diagnostic tools—a two‐step workflow based on plasma Aβ42 and *p*‐tau217 quantification. Patients will be recruited from multiple French memory clinics based on cognitive complaints or a family history of dementia. They will undergo baseline evaluations using state‐of‐the‐art tools, including a comprehensive neuropsychological assessment, MRI imaging, gut microbiota analysis, blood‐based large‐scale proteomic analysis (Nulisa), and sleep evaluation. Longitudinal monitoring using digital tools will enable the detection of cognitive decline, even remotely, and facilitate timely referral for a full memory clinic evaluation. This approach aims to initiate disease‐modifying treatments at the earliest possible stage, if appropriate.

**Result:**

Our main goal is to establish a cohort of amyloid‐positive patients who have been screened using the most up‐to‐date plasma biomarkers. By evaluating these patients longitudinally, we aim to treat them at the earliest possible stage when they begin to exhibit cognitive decline. Additionally, we hope to identify biological parameters—whether individually, in combination, or through their progression—that can best predict cognitive decline, thereby enabling us to identify the population most likely to benefit from treatments.

**Conclusion:**

Not all amyloid‐positive individuals will exhibit cognitive decline or progress to Alzheimer's disease. Amyloidopathy alone cannot be used to justify initiating disease‐modifying treatments, especially given their significant side effects. Our project aims to provide new insights into the biological parameters that can predict cognitive decline, ultimately supporting more informed and patient‐centered clinical decisions.

